# Treatment of Granuloma Annulare With Pentoxifylline and Oral Vitamin E: A Case Series

**DOI:** 10.7759/cureus.18151

**Published:** 2021-09-21

**Authors:** Timothy E Holland, Lauren W Holland, Austinn C Miller, William E Freeman

**Affiliations:** 1 United States Air Force (USAF) Medical Service, United States Air Force, Falls Church, USA; 2 Dermatology, Houston Medical Center, Warner Robins, USA; 3 Dermatology, Baylor College of Medicine, Houston, USA

**Keywords:** granuloma annulare, pentoxifylline, vitamin e, disseminated granuloma annulare, granulomatous disorder

## Abstract

Granuloma annulare (GA) is a difficult-to-treat granulomatous skin disorder characterized by multiple annular, raised, and erythematous lesions. While numerous therapies have been proposed, there is currently no clear gold standard of treatment.

We present a series of five patients with biopsy-proven generalized GA who were treated with pentoxifylline and oral vitamin E. Each patient in this series had at least a one-year history of GA refractory to other treatments. After three months of treatment, four of the five patients demonstrated objective improvement in their lesions through before and after clinical photographs. No patients suffered any adverse events on this treatment regimen.

As our series has demonstrated modest benefits in concurrence with previously published studies, we recommend combination therapy with pentoxifylline and vitamin E as a strong contender for first- or second-line treatment for generalized GA. Pentoxifylline and vitamin E are advantageous for their tolerability, cost-effectiveness, minimal drug interactions, and convenience.

## Introduction

Granuloma annulare (GA) is a notoriously difficult-to-treat granulomatous skin condition characterized by annular, raised, erythematous papules, which often coalesce into plaques. Although considered benign, GA with its conspicuous lesions may persist for years and is very often distressing for the afflicted patient. There are several distinct clinical patterns of GA, including localized GA, generalized GA (GGA), subcutaneous GA, and perforating GA. While localized GA, the most common subtype, tends to resolve spontaneously, GGA may persist for years and is often more difficult to manage.

Granuloma annulare has easily recognized clinical features, but there is an incomplete understanding of its pathogenesis and a significant lack of effective management strategies. Multiple treatments have been proposed for GGA: dapsone, Plaquenil, topical calcineurin inhibitors, topical corticosteroids, psoralen plus ultraviolet A (PUVA), and antibiotics isotretinoin and allopurinol, among many others [1-3]. All of these have varying levels of efficacy, with no clear gold standard of treatment.

The literature describes several case reports and series with remarkable success using pentoxifylline, a phosphodiesterase inhibitor typically indicated for claudication-associated peripheral arterial disease [4-7]. Oral vitamin E has also been noted to effectively treat GGA [8-9]. Both of these therapies are cost-effective, convenient, well-tolerated, and have minimal drug interactions. We present a series of five patients with GGA treated simultaneously with pentoxifylline and oral vitamin E.

## Case presentation

Five patients of an outpatient, private practice dermatology office were studied for this case series. Eligibility criteria included biopsy-proven GGA for at least one year and failure of at least three previous therapies. In our study, GGA is defined as 10 or more lesions covering at least three separate anatomical areas. The patients were started on 400 milligrams of pentoxifylline twice daily in addition to 400 IU of oral Vitamin E daily. Each case was followed for three months, and efficacy was determined by subjective benefit reported by the patients and comparison of pre and post-treatment clinical photographs. Cases are summarized in Table 1.

**Table 1:** Summary of cases

Case	Sex	Age	Comorbidities	Previous Therapies	Subjective Response	Objective Response
1	F	74	Myasthenia Gravis, Diabetes Mellitus Type 2, Hyperlipidemia, Hypertension	Triamcinolone 0.5% cream, Pimecrolimus 1% cream, Desoximometasone 0.25% cream, Flurandrenolide tape (4 mcg/cm^2^)	“Mild” improvement	Mild response - Lesions over upper extremities lighter in color
2	M	66	Diabetes Mellitus Type 2, Hyperlipidemia, Hypertension, Melanoma	Hydrocortisone 2.5% cream, Tacrolimus 0.5% cream, Clobetasol 0.05% ointment	No improvement	Mild response - Fewer lesions over lower extremities, lesions over upper extremities decreased in size and lighter in color.
3	F	67	Breast Cancer treated with mastectomy, Hypothyroidism, Psoriasis	Fluocinonide 0.05% cream, Clobetasol 0.05% cream, Tretinoin 0.01% gel, Prednisone 20mg x 14 days, Halobetasol 0.01% lotion, Rifamycin 600mg + Ofloxacin 400mg + Minocycline 100mg once monthly (ROM therapy), Plaquenil 200mg	“Excellent” improvement	Significant response - lesions considerably lighter, decreased number and size of lesions over upper and lower extremities
4	F	64	Diabetes Mellitus Type 2, Hyperlipidemia, Hypertension, Hypertriglyceridemia	Halobetasol 0.05% cream, Clobetasol 0.05% cream, ROM therapy, Intralesional triamcinolone 4% injections, calcipotriene 0.005% foam	“Moderate” improvement	Moderate response - Lesions over upper extremities decreased in size and number, lesions lighter in color
5	M	61	Coronary Artery Disease, Hypertension, Prostate Cancer	Betamethasone dipropionate 0.05% cream, Calcipotriene 0.005% foam, Dapsone 25mg, Plaquenil 200mg	“Mild” improvement	No response

Case 1

Patient 1 is a 74-year-old woman with comorbid hypertension, diabetes mellitus type 2, hyperlipidemia, and myasthenia gravis. She was diagnosed with GGA 5 years prior and had experienced very limited success with topical pimecrolimus 0.1% cream, triamcinolone 0.5% cream, desoximetasone 0.25% cream, and flurandrenolide tape. After three months of therapy with pentoxifylline and oral vitamin E, she noted mild benefits and reported her lesions were lighter in color as compared with using only topical therapies. Clinical photographs demonstrated some benefit, particularly in her bilateral distal upper extremities.

Case 2

Patient 2 is a 66-year-old male with comorbid hypertension, diabetes mellitus type 2, and hyperlipidemia. He was diagnosed with GGA one year prior and had experienced very limited success with topical hydrocortisone 2.5% cream, tacrolimus 0.5% cream, and clobetasol 0.05% ointment. After three months of therapy with pentoxifylline and vitamin E, the patient denied any benefit. However, clinical photographs demonstrated a decrease in the size and number of lesions on his bilateral lower extremities.

Case 3

**Figure 1:**
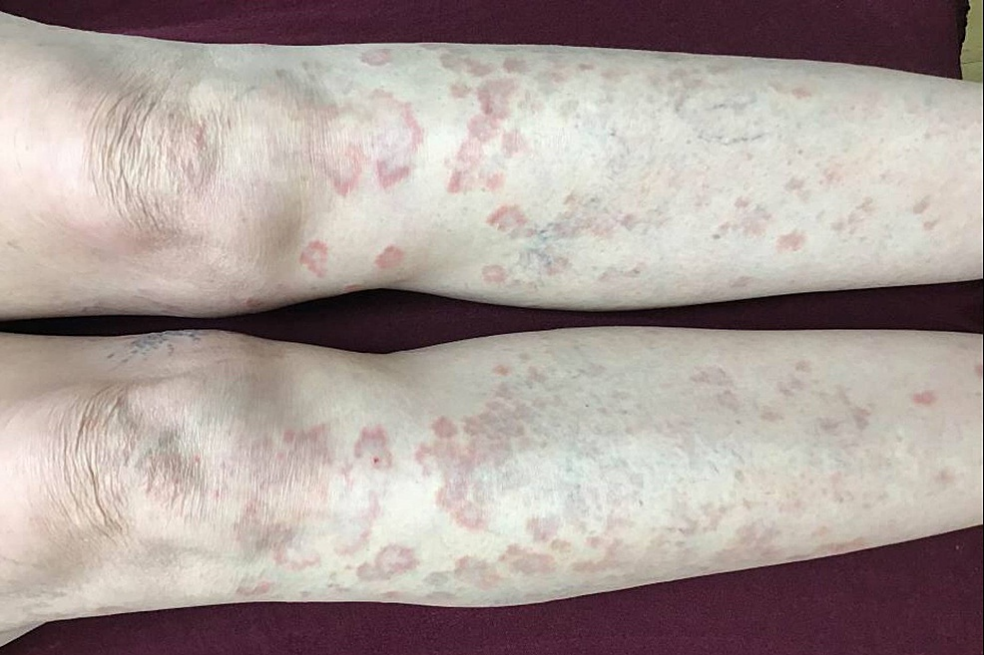
Clinical photograph of anterior bilateral lower extremities of case 3 before three months of treatment with pentoxifylline and vitamin E

**Figure 2:**
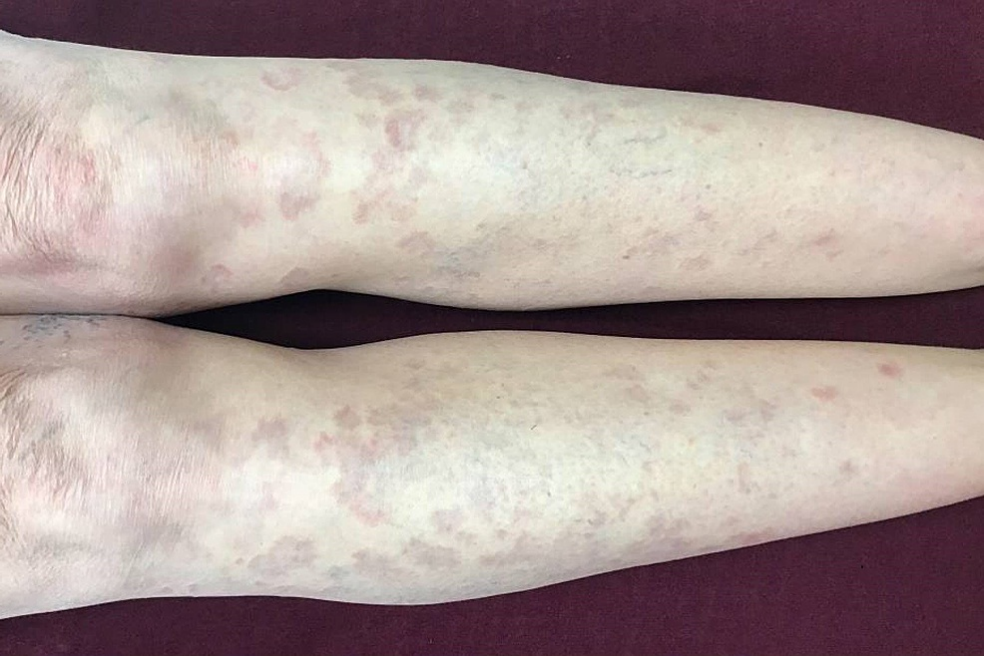
Clinical photograph of anterior bilateral lower extremities of case 3 after three months of treatment with pentoxifylline and vitamin E

Patient 3 is a 67-year-old female with comorbid hypothyroidism, remitting breast cancer, and psoriasis. Her GGA had been present for just over two years, with limited to no relief on fluocinonide 0.05% cream, clobetasol 0.05% cream, tretinoin 0.01% gel, halobetasol 0.01% lotion, monthly Rifampicin 600 mg, Ofloxacin 400 mg, and Minocycline 100 mg (ROM therapy) for six months, and a two-week course of prednisone 20 mg daily. The patient noted mild clearing on ROM therapy but otherwise had no significant relief with other treatment. After three months of therapy with pentoxifylline and vitamin E, she noted a substantial improvement in her GGA lesions, which was consistent with her clinical photographs (Figures 1-2). Of note, this patient is the only one in our series without a diagnosis of diabetes mellitus type 2.

Case 4

**Figure 3:**
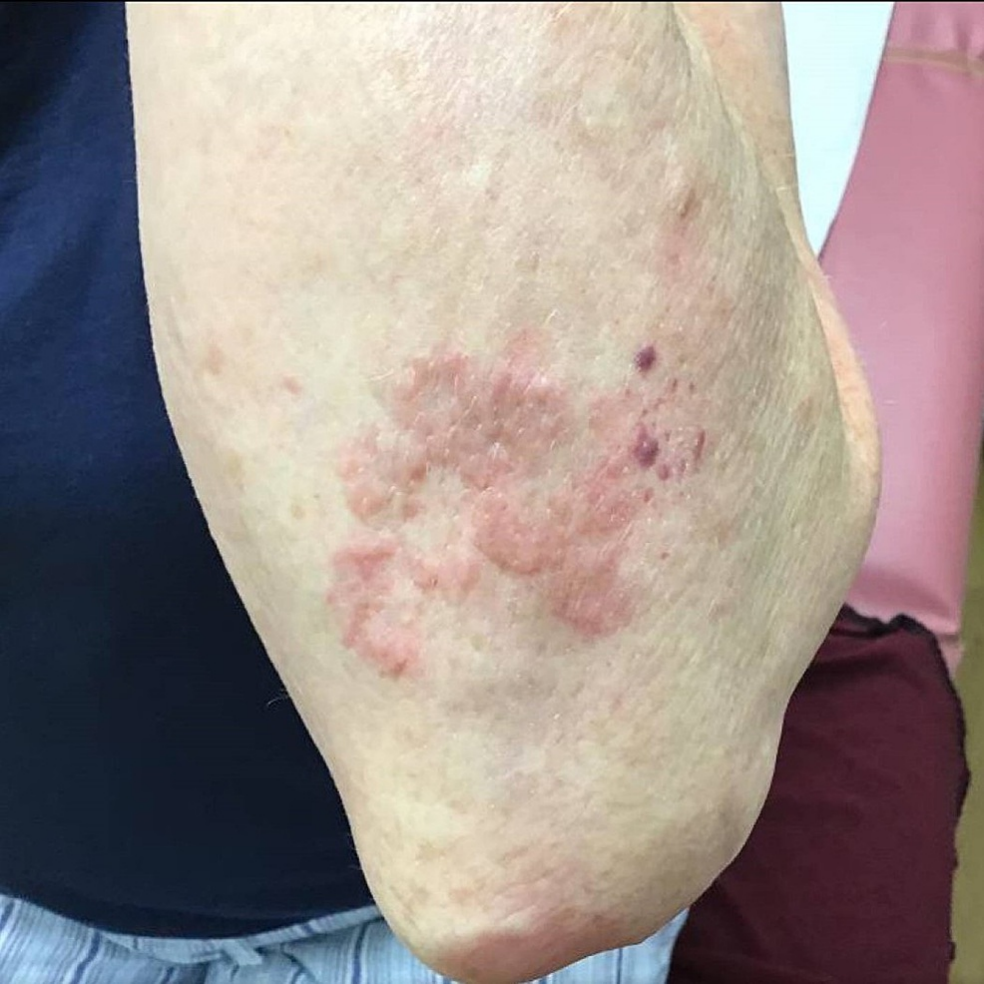
Clinical photograph of the posterior left elbow of case 4 before three months of treatment with pentoxifylline and vitamin E

**Figure 4:**
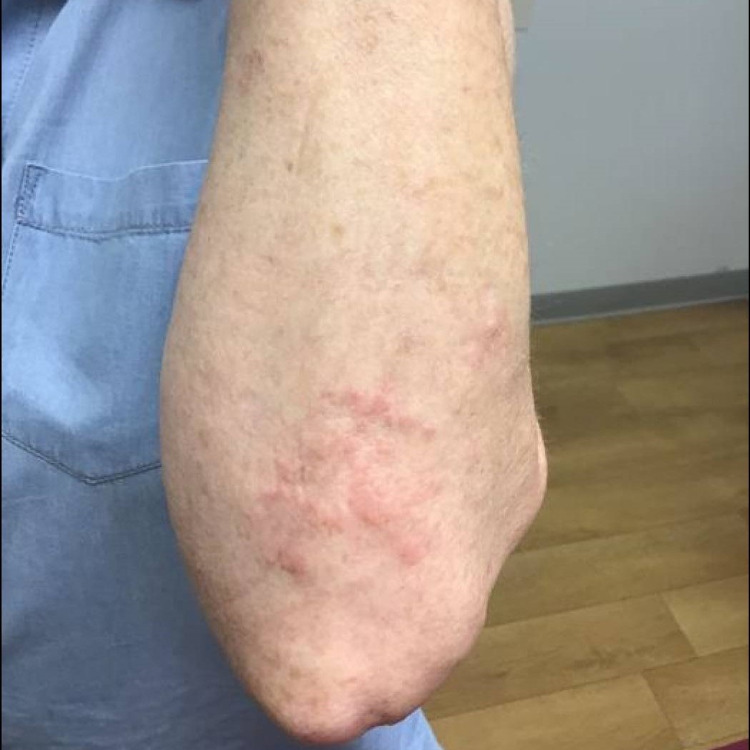
Clinical photograph of the posterior left elbow of case 4 after three months of treatment with pentoxifylline and vitamin E

Patient 4 is a 64-year-old female with comorbid hypertension, diabetes mellitus type 2, hyperlipidemia, and hypertriglyceridemia. She had a five-year history of GGA with limited success on halobetasol 0.05% cream, clobetasol 0.05% cream, ROM therapy, intralesional triamcinolone 4% injections, and calcipotriene 0.005% foam. She denied substantial improvement after three months of pentoxifylline and vitamin E therapy, although clinical photographs demonstrated a reduction in erythema and the size of her lesions (Figures 3-4).

Case 5

Patient 5 is a 61-year-old male with comorbid hypertension, diabetes mellitus type 2, and hyperlipidemia. He had an eight-year history of GGA and experienced some relief previously with topical betamethasone 0.05% cream, calcipotriene 0.005% foam, dapsone 25 mg, and Plaquenil 200 mg. After three months of treatment with pentoxifylline and oral vitamin E, he noted a halting of his disease progression, no evolving or new lesions, and signs of regression through fading lesions. However, our clinical photographs did not demonstrate improvement.

## Discussion

This case series demonstrated a combination of two oral therapies for the treatment of GGA in five patients from an outpatient dermatology clinic. Four (80%) patients noted subjective improvement in their lesions. Objective improvement was seen in clinical photographs before and after therapy in four (80%) patients. No patients reported any adverse events on therapy.

**Figure 5:**
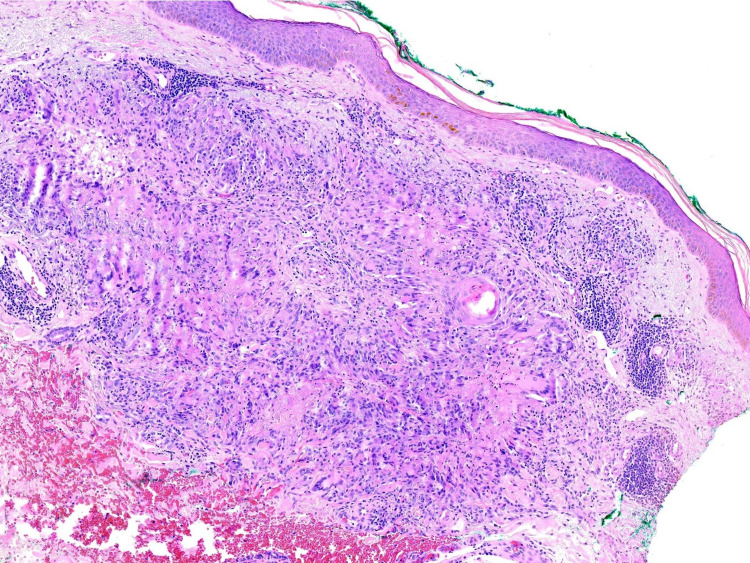
Punch biopsy demonstrating granulomatous inflammation with abundant histiocytes in the superficial and mid dermis in addition to perivascular lymphocytes H&E 40X

**Figure 6:**
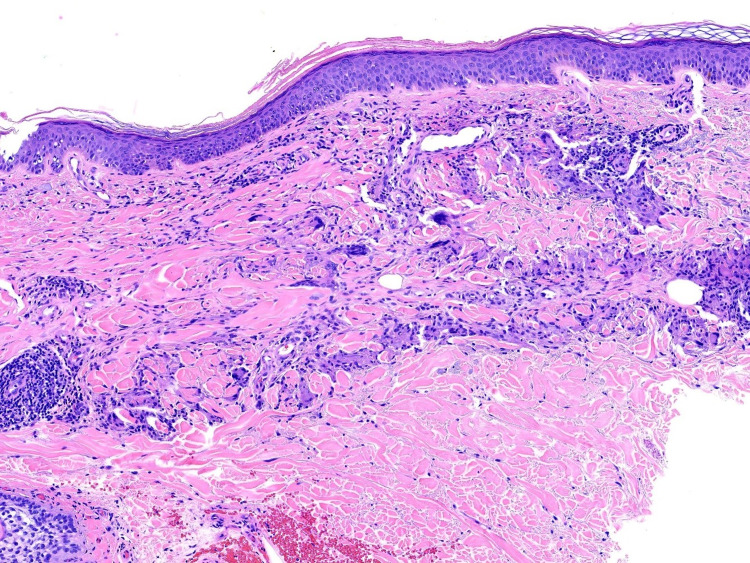
Classic appearance of palisading granuloma surrounding central necrobiosis H&E 100x

Histologically, GA demonstrates the pathognomonic features of mucin deposition along with palisading histiocytes and lymphocytes surrounding a central zone of necrobiotic collagen in the upper and middle dermis (Figures 5-6) [10]. 

While the complete pathophysiology for GA is not yet elucidated, the disease appears to be the result of a delayed-type hypersensitivity reaction mediated by CD3+ TH1 cells. Cytokines such as tumor necrosis factor (TNF)-alpha, interleukin 2 (IL-2), and others have been revealed as potential factors [10]. Several disease states are commonly associated with GGA, including diabetes mellitus type 2 (DMT2), solid malignancies, and hypothyroidism, although the former of these is often debated as a true association. Nevertheless, patients afflicted with DMT2 often had GA that was more chronic compared with those without [10]. This leads to a theory that microvascular damage may promote the formation of granulomas, thereby contributing to the development of GA.

Although there is an abundance of studies on treating GGA, there is a lack of high-quality evidence to support a ‘gold standard therapy’ due to variable treatment success. In one review by Lukas et al. [1], a total of 91 articles (a vast majority of which were case reports or case series) suggested 26 unique treatment modalities with varying degrees of success. Decisions for therapy are based on many factors: efficacy, cost, availability, convenience, adverse events, drug interactions, and patient compliance. Based on these criteria and a review of the literature, we chose combination therapy with pentoxifylline and oral vitamin E.

Pentoxifylline is a xanthine derivative, which is a non-selective inhibitor of phosphodiesterase. Downstream effects include raising intracellular cyclic adenosine monophosphate (cAMP) and TNF-alpha suppression. TNF-alpha is a frequently studied cytokine and has relevance to GA, as it is implicated in granuloma formation. In addition, pentoxifylline improves red blood cell deformability, which decreases blood viscosity and improves tissue perfusion [11]. With many of our patients having comorbid vascular disease, increasing tissue perfusion through pentoxifylline may have contributed to their improvement. The most commonly reported adverse events related to pentoxifylline are nausea and vomiting, but otherwise, this medication is generally well-tolerated. Pentoxifylline may enhance the effects of anti-hypertensives and some anti-glycemic medications, so for those patients, blood pressure and glucose monitoring should be considered. There is a potential risk of increased bleeding in patients taking warfarin or antiplatelet agents, so caution should be used. Finally, as a xanthine derivative, pentoxifylline may also increase levels of theophylline. As this medication is notorious for a narrow therapeutic index, pentoxifylline should generally be avoided in these patients [12].

Tocopherol, or vitamin E, exerts its mechanism of action by sequestering free radicals. A study by Khatami et al. found that supplementation with oral vitamin E decreased serum biomarkers of several inflammatory mediators, including TNF, metalloproteinases, and malondialdehyde (a marker of oxidative stress) [13]. Another benefit of vitamin E is patient preference, as many patients prefer treatment that is more ‘natural’ and familiar to them. There is a potential risk of inducing vitamin K deficiency with very large doses (and thus should be avoided in patients with a known deficiency), but otherwise, it is not associated with any known adverse events [14].

## Conclusions

While the management of GGA is often pursued, it remains important to acknowledge that this disease is benign, self-limited, and most often asymptomatic for the afflicted patient. Therefore, treatment, if sought, should have minimal to no adverse events, be cost-effective, and be readily available to the patient. Our series demonstrates that pentoxifylline and oral vitamin E meet these criteria, and in concordance with previously published studies, show modest benefit for patients with GGA. Larger clinical trials with appropriate blinding and controls are necessary to measure a true clinical benefit. However, the allure of an affordable, well-tolerated, and convenient treatment should make this combination a strong contender for first-line therapy.

## References

[1] Lukács J, Schliemann S, Elsner P (2015). Treatment of generalized granuloma annulare - a systematic review. J Eur Acad Dermatol Venereol.

[2] Piette EW, Rosenbach M (2016). Granuloma annulare. Pathogenesis, disease associations and triggers, and therapeutic options. J Am Acad Dermatol.

[3] Wang J, Khachemoune A (2018). Granuloma annulare: a focused review of therapeutic options. Am J Clin Dermatol.

[4] Nambiar KG, Jagadeesan S, Balasubramanian P, Thomas J (2017). Successful treatment of generalized granuloma annulare with pentoxifylline. Indian Dermatol Online J.

[5] Rubel DM, Wood G, Rosen R, Jopp-McKay A (1993). Generalised granuloma annulare successfully treated with pentoxifylline. Australas J Dermatol.

[6] Visconti MJ, Ashack KA, Ashack RJ (2021). Granuloma annulare: strengthening potential associations and pentoxifylline as a therapeutic option. J Dermatolog Treat.

[7] Wong GN, Wee E, Tam M, Kelly R (2019). Pentoxifylline as a treatment for granuloma annulare. Australas J Dermatol.

[8] Poppe H, Poppe LM, Goebeler M, Trautmann A (2013). Treatment of disseminated granuloma annulare with oral vitamin E: 'primum nil nocere'. Dermatology.

[9] Smith KJ, Norwood C, Skelton H (2002). Treatment of disseminated granuloma annulare with a 5-lipoxygenase inhibitor and vitamin E. Br J Dermatol.

[10] Keimig EL (2015). Granuloma Annulare. Dermatol Clin.

[11] Marques LJ, Zheng L, Poulakis N, Guzman J, Costabel U (1999). Pentoxifylline inhibits TNF-alpha production from human alveolar macrophages. Am J Respir Crit Care Med.

[12] Annamaraju P, Baradhi KM (2021). Pentoxifylline. http://www.ncbi.nlm.nih.gov/books/NBK559096/.

[13] Khatami PG, Soleimani A, Sharifi N, Aghadavod E, Asemi Z (2016). The effects of high-dose vitamin E supplementation on biomarkers of kidney injury, inflammation, and oxidative stress in patients with diabetic nephropathy: a randomized, double-blind, placebo-controlled trial. J Clin Lipidol.

[14] Booth SL, Golly I, Sacheck JM, Roubenoff R, Dallal GE, Hamada K, Blumberg JB (2004). Effect of vitamin E supplementation on vitamin K status in adults with normal coagulation status. Am J Clin Nutr.

